# An ultrahigh sensitivity acoustic sensor system for weak signal detection based on an ultrahigh-*Q* CaF_2_ resonator

**DOI:** 10.1038/s41378-023-00540-0

**Published:** 2023-05-17

**Authors:** Tong Xing, Enbo Xing, Tao Jia, Jianglong Li, Jiamin Rong, Li Li, Sicong Tian, Yanru Zhou, Wenyao Liu, Jun Tang, Jun Liu

**Affiliations:** 1 Key Laboratory of Dynamic Testing Technology, School of Instrument and Electronics, North University of China, Taiyuan, 030051 China; 2 School of Semiconductors and Physics, North University of China, Taiyuan, 030051 China; 3 Shanxi Key Laboratory of Advanced Semiconductor Optoelectronic Devices and Integrated Systems, Jincheng, 048026 China; 4 State Key Laboratory of Luminescence and Applications, Changchun Institute of Optics, Fine Mechanics and Physics, Chinese Academy of Sciences, Changchun, 130033 China

**Keywords:** Optical sensors, Micro-optics

## Abstract

Acoustic sensors with ultrahigh sensitivity, broadband response, and high resolution are essential for high-precision nondestructive weak signal detection technology. In this paper, based on the size effect of an ultrahigh-quality (*Q*) calcium fluoride (CaF_2_) resonator, a weak acoustic signal is detected by the dispersive response regime in which an acoustic, elastic wave modulates the geometry and is converted to a resonance frequency shift. Through the structural design of the resonator, the sensitivity reaches 11.54 V/Pa at 10 kHz in the experiment. To our knowledge, the result is higher than that of other optical resonator acoustic sensors. We further detected a weak signal as low as 9.4 µPa/Hz^1/2^, which greatly improved the detection resolution. With a good directionality of 36.4 dB and a broadband frequency response range of 20 Hz–20 kHz, the CaF_2_ resonator acoustic sensing system can not only acquire and reconstruct speech signals over a long distance but also accurately identify and separate multiple voices in noisy environments. This system shows high performance in weak sound detection, sound source localization, sleep monitoring, and many other voice interaction applications.

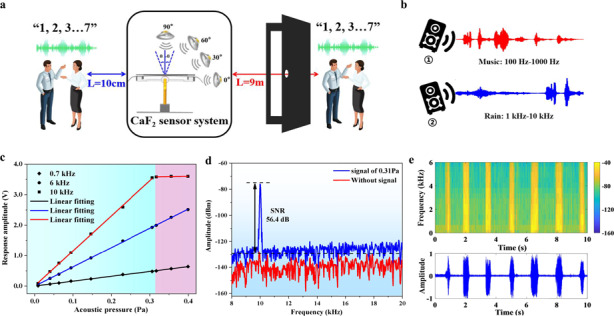

## Introduction

Acoustic sensing based on photon detection technology converts optical signals into electrical signals using high-sensitivity photon detectors, which can achieve a fast response when external acoustic signals are coupled to form acoustic-optical-electrical sensing logic^1–4^. The extremely low noise and high sensitivity provide the capacity for weak acoustic signal detection^5,6^. Combined with the advantages of high detection efficiency and time accuracy, it has attracted wide research interest in the last decade and has shown great potential in applications such as industrial nondestructive testing^7^, natural disaster warning^8^, medical and health diagnosis^9^, and photoacoustic imaging^10^. The ultrahigh quality factor (*Q*) and the small mode volume of the whispering-gallery-mode (WGM) optical resonator significantly enhance the light–matter interaction^11–14^. Importantly, the ultrahigh *Q* corresponds to the extremely narrow transmission spectrum, and higher solution resolution can be obtained either by detecting frequency shift or transmission spectrum broadening, theoretically making it an excellent platform for ultrahigh sensitivity acoustic sensing^15–17^. In addition, compared with the electrical sensor of the traditional piezoelectric effect, it also has the advantage of anti-electromagnetic interference^18^.

From the perspective of physical mechanisms, acoustic sensing based on WGM resonators mainly originates from two mechanisms: dispersive coupling response and dissipative coupling response^19,20^. The dispersive coupling response is that the acoustic wave modulates the refractive index and geometric morphology of the resonator through mechanical effects, resulting in a shift in the resonance frequency of the resonator, and its sensitivity reaches 280 mV/Pa^21^. The dissipative coupling response is the change in the coupling conditions of the resonator through acoustic wave modulation, leading to a change in the coupling loss, which broadens or narrows the transmission spectrum linewidth. A noise equivalent pressure (NEP) as low as 0.81 Pa at 140 kHz in the air has been experimentally demonstrated^22^. From the perspective of detection methods, the method of using mechanical frequency as a reference through the cavity optomechanical effect can effectively suppress the noise, and the NEP at the ultrasonic frequency can reach 8–300 μPa/Hz^1/2^. However, the sensor can only achieve the best sensitivity in narrow frequency windows near each mechanical resonance, which limits the dynamic response range^23^. Moreover, the inherent optomechanical coupling coefficient «1 in the resonator is a problem for achieving high sensitivity (V/Pa). In contrast, high sensitivity can be obtained by direct detection from the optical frequency domain, but it is also limited by the random walk of the resonance frequency limited by shot noise^24^.

High-quality speech signal acquisition and reconstruction^25–28^, especially for accurate speech recognition in noisy backgrounds, requires acoustic sensors combined with ultrahigh sensitivity, broadband response, and low noise. In particular, acoustic sensors have a high response in the low-sound band, so there is an urgent need to develop acoustic sensing technology with ultrahigh sensitivity over a wide frequency range. Among all WGM resonators, the calcium fluoride (CaF_2_) crystalline resonator has unique advantages^29–31^. On the one hand, a CaF_2_ resonator with *Q* > 10^11^ at 1550 nm has been reported in the experiment^32^, which theoretically improves the acoustic sensitivity by several orders of magnitude. By further flexibly adjusting the structure of the CaF_2_ resonator, low NEP, which is conducive to the detection of weak acoustic signals, can be obtained. On the other hand, the CaF_2_ resonator supports multiple modes with different *Q*, leading to fast switching with ultrahigh sensitivity and a wide dynamic response range by frequency locking technology^21^.

In this paper, based on the ultrahigh-*Q* CaF_2_ resonator, the weak acoustic signal is detected by the dispersive response regime in which the acoustic, elastic wave modulates the geometry and is converted to a resonance shift. Combined with the actual processing technology, a CaF_2_ resonator with a radius of 5.0 mm and a thickness of 0.1 mm is obtained by structural design and verification. In the experiment, using the frequency locking technique, the sensitivity reaches 11.54 V/Pa at 10 kHz frequency when *Q* is selected as 1.02 × 10^8^. To the best of our knowledge, the result is higher than that of other optical resonator acoustic sensors. Meanwhile, the minimum detectable acoustic pressure level of the system is as low as 9.4 µPa/Hz^1/2^, which greatly improves the detection resolution. With a good directionality of 36.4 dB and broadband frequency response range of 20 Hz–20 kHz, the sensor system can not only achieve long-distance (9 m) speech signal acquisition and reconstruction with the wall as an obstacle but also accurately identify and separate multiple voices in noisy environments. This system performs well with regard to weak sound detection, sound source localization, sleep monitoring, and many other voice interaction applications.

## Theoretical analysis and discussion

**Fig. 1:**
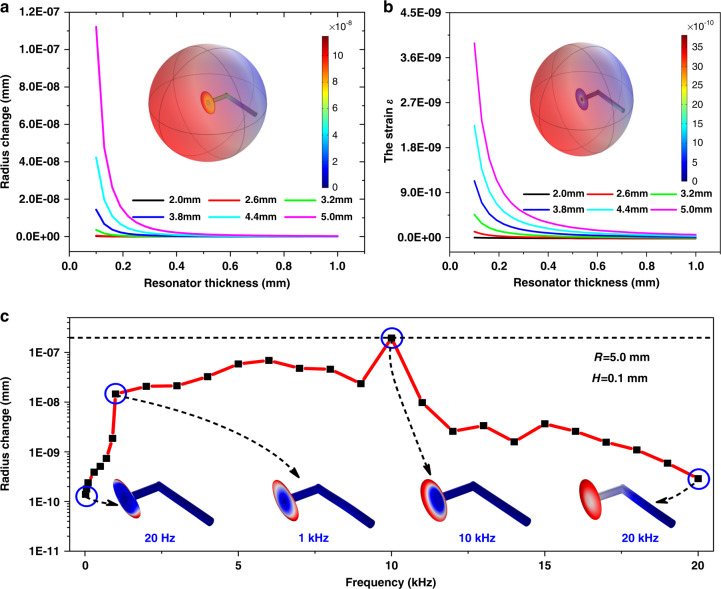
Structural optimization and frequency response by simulation. **a**, **b** The variation in the radius and strain at different radii and thicknesses. The insets show the change in the radius and strain when the radius is 5.0 mm, and the thickness is 0.1 mm at 1 Pa. **c** The frequency response characteristics of the 7-shaped CaF_2_ resonator system

In the CaF_2_ resonator, light is coupled into the resonator and circulates along the resonator’s circumference. In the sensing system with a CaF_2_ resonator as the sensitive unit, the acoustic pressure causes mechanical deformation of the resonator, and the radius and effective refractive index of the resonator change, resulting in a resonance shift. This is different from the conventional mechanism of enhanced optomechanical interaction between the optical radiation field and the mechanical vibration modes by confining the optical field within the resonator^23^. To obtain an acoustic sensor with vector properties, a CaF_2_ resonator with a 7-shaped structure is fabricated (Supplementary Figs. S15 and S16). The resonator structure is optimized by the simulation to obtain the optimal structural parameters. The basic material properties are shown in Supplementary Table S1. By using finite element method (FEM) simulation (Supplementary Tables S2 and S3), when an acoustic pressure of 1 Pa is applied to the upper surface of the 7-shaped CaF_2_ resonator, the changes in radius and strain at different radii (*R*) and thicknesses (*H*) are analyzed in Fig. 1a, b, respectively. With increasing *R* and decreasing *H*, the variations in the resonator radius and strain increase rapidly. When *R* is 5.0 mm, and *H* is 0.1 mm, the variations in the radius and strain are the largest, which are 1.13 × 10^−7^ mm/Pa and 3.97 × 10^−9^ Pa^−1^, respectively. The influence of the strain is negligible since the variation in the radius is an order of magnitude greater than the variation in the strain^21^. Thus, the shaped variable of the sensor system can be improved remarkably by thinning the CaF_2_ resonator with a larger radius.

To analyze the frequency response characteristics of the CaF_2_ resonator, the simulation of the displacement at different resonance frequencies is calculated by the FEM and shown in Fig. 1c. In the 20 Hz–10 kHz frequency band, the displacement shows an overall increasing trend, and the maximum displacement is 10 kHz. After 10 kHz, the displacement generally tends to decrease as it moves away from the mechanical resonance frequency. It can be clearly seen that the response appears to have an envelope shape, indicating a higher response effect in this region (1–10 kHz). The corresponding displacement pattern diagram is shown in Supplementary Fig. S3. It is worth noting that the frequency response can continue up to 20 kHz with broadband response characteristics, although the response of this frequency band is not as strong. Moreover, the frequency response is adjustable. If the structure of the CaF_2_ resonator is changed, the corresponding frequency response is also different. Therefore, it can meet the requirements of different applications.

Using coupled-mode theory, the optical mode amplitude *a* is expressed by

1 $$\frac{da(t)}{dt}=(i(\varDelta +Gsin({\omega }_{m}t))-\frac{\kappa }{2})a(t)+\sqrt{{\kappa }_{e}}{a}_{in}$$

where Δ = *ω*_*L*_ − *ω*_*r*_ is the detuning of the laser from the optical resonance, *G* represents the resonance shift due to the acoustic pressure, *κ* is the overall intensity decay rate, *κ*_*e*_ represents the input coupling losses, *a*_in_ is the input optical field into the resonator, and *ω*_m_ is the acoustic frequency.

According to the resonance wavelength equation, the resonance wavelength shift becomes

2 $$\frac{d{\lambda }_{r}}{dP}=\frac{{\lambda }_{r}}{dP}\frac{d{\lambda }_{r}}{{\lambda }_{r}}\approx \frac{{\lambda }_{r}}{dP}\frac{dR}{R}$$

The sensitivity of the CaF_2_ resonator acoustic sensor system can be defined as

3 $$S=\frac{dT}{dP}=\frac{d{\lambda }_{r}}{dP}\frac{dT}{d{\lambda }_{r}}=\frac{{\lambda }_{r}}{dP}\frac{dR}{R}\frac{dT}{d{\lambda }_{r}}$$

where *T* is the transmission, *P* is the acoustic pressure, and *λ*_*r*_ is the resonance wavelength. The first term (dλ_*r*_/d*P*) is the acoustic wave-induced resonance wavelength shift, which can be divided into (*λ*_*r*_/d*P*) and (d*R*/*R*). (d*R*/d*P*)(1/*R*) represents the change rate of the CaF_2_ resonator radius under acoustic pressure. The second term d*T*/d*λ*_*r*_ is the slope of the resonance spectrum, which is related to the *Q* and the photodetector (PD). The saturation power of the PD is 360 μW, and the conversion gain is 10^4^ V/W.

**Fig. 2:**
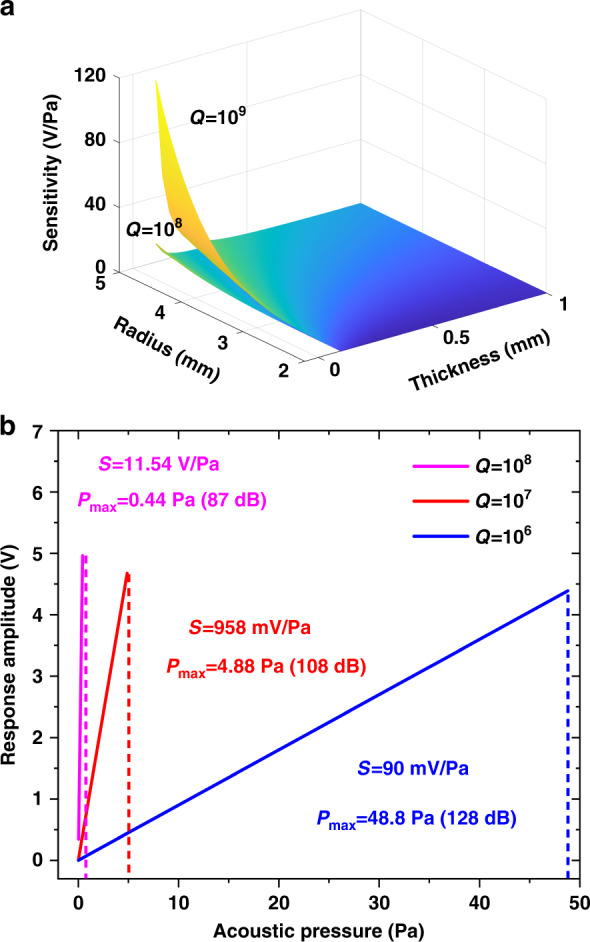
Theoretical performance evaluation of the sensor system. **a** The theoretical sensitivity at different radii and thicknesses. **b** The relationship between the sensitivity and the maximum detectable acoustic pressure (*P*_max_) at different *Q*

Different resonator sizes correspond to different sensitivities, so we analyze the effect of the size on the response sensitivity, which is illustrated in Fig. 2a. When the radius of the CaF_2_ resonator is greater than 4.0 mm, and the thickness is less than 0.2 mm, the curve changes rapidly, representing that this is the most sensitive region. In addition, the changes in other parts are very small, and the sensitivity is relatively low. The higher the *Q* of the resonator is, the greater the sensitivity at the same size. When *Q* is selected as 10^8^, the sensitivity can be as high as 11 V/Pa. Moreover, when *Q* is selected as 10^9^, the sensitivity can exceed 100 V/Pa. Increasing the resonator radius improves sensitivity, but it also limits the maximum detectable acoustic pressure, as shown in Fig. 2b. The maximum detectable acoustic pressure is calculated from the relationship between the full width at the half maximum (∆*λ*) and the shift in the resonance wavelength. When *Q* is selected as 10^8^, the sensitivity can be as high as 11.54 V/Pa, but the maximum detection acoustic pressure (*P*_max_) is only 0.44 Pa (87 dB). When *Q* is selected as 10^6^, the sensitivity is only 90 mV/Pa, but the *P*_max_ can be 48.8 Pa (128 dB). The *Q* of the CaF_2_ resonator prepared by our experiment can reach 10^9^, but the *P*_max_ is only 0.05 Pa (68 dB). Therefore, the sensitivity and *P*_max_ for *Q* = 10^9^ are not plotted in Fig. 2b. In conclusion, the larger the radius of the CaF_2_ resonator is, the thinner the thickness, and the correspondingly higher the sensitivity is with the same *Q* factor. The larger *Q* is, the higher the sensitivity, and the smaller the *Q* is, the wider the dynamic response range.

## Experimental setup and method

**Fig. 3:**
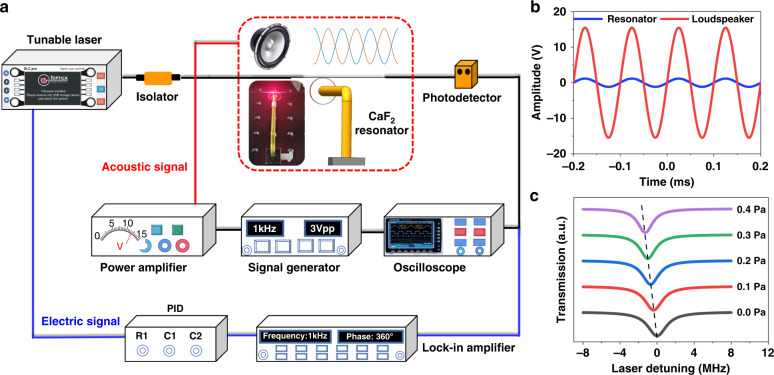
Performance characterization of the CaF_2_ resonator acoustic sensor. **a** Schematic measurement setup of the CaF_2_ resonator acoustic sensor system. **b** The voltage signal received by the loudspeaker and the resonator. **c** The shift of the resonance spectrum changes with the acoustic pressure (0.0–0.4 Pa)

Figure 3a schematically shows the 7-shaped CaF_2_ resonator acoustic sensor system to characterize the performance, which includes a tunable laser, an isolator, a PD, a signal generator (SG), a power amplifier (PA), an oscilloscope (OSC), a PID controller and a lock-in amplifier (LIA). The tunable laser has a center wavelength of 1550 nm, and it is scanned with a triangular wave at a frequency of 10 Hz and an amplitude of 3 V. The isolator blocks the light returning to the laser. The CaF_2_ resonator with a radius of 5.0 mm and a thickness of 0.1 mm is fabricated by a single-point diamond cutting and mechanical polishing method. The radius of the tapered fiber is 1.8 μm (Supplementary Fig. S2), and the tapered length is 2.0 cm to form evanescent field coupling. The coupling between the tapered fiber and the resonator is covered with a transparent cover to reduce external environmental interference. The light from the tapered fiber is connected to the PD for conversion between optical and electrical signals and then connected to the OSC for data acquisition and processing. In the experiment, the tapered fiber is attached to the surface of the CaF_2_ resonator, which not only avoids the influence of environmental fluctuation noise but also eliminates the influence of the dissipative response regime (Supplementary Fig. S1).

The SG outputs sinusoidal signals with different frequencies and amplitudes to the PA, which outputs voltage signals to control the frequency and amplitude of the acoustic source. The source signal is connected to the OSC along with the voltage signal from the resonator. Since the loaded acoustic wave propagates in the gas medium, the loudspeaker is located 10 cm away from the resonator sensing system, and the acoustic wave loses energy in the process of propagation. By comparison, the voltage signal received by the resonator is 13.4 times lower than that of the loudspeaker source signal, as shown in Fig. 3b. According to the input–output relationship, the normalized transmission changes with the acoustic pressure, as shown in Fig. 3c. (Supplementary Fig. S7). Traditional detection by frequency shift is affected by the environment. To improve the detection accuracy, an acoustic signal is detected by locking the laser frequency on the resonance point of the CaF_2_ resonator. According to the relationship between the demodulated curve and the resonance curve, the demodulated signal is used as an error signal for feedback control of the laser frequency to achieve frequency locking through the PID controller. Therefore, the resonance frequency shift corresponds to the offset of the response amplitude related to the locking point.

## Sensitivity and minimum detectable acoustic pressure

**Fig. 4:**
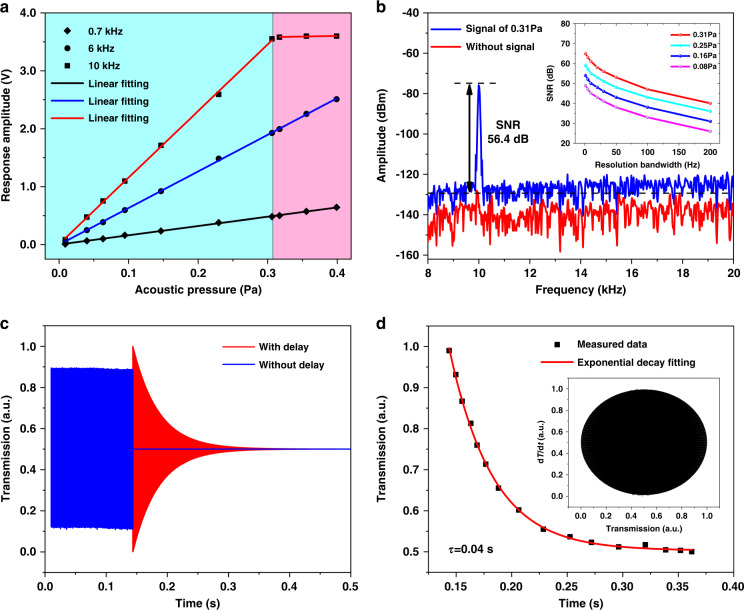
Evaluation of the performance of the sensor device. **a** Variation in the response amplitude with the acoustic pressure at different frequencies with errors. **b** Frequency domain spectrum of an acoustic signal at 10 kHz and without signal. The inset shows the relationship between *SNR* and *BW* at different *P*_applied_. **c** The delay response curve when the loaded signal disappears. **d** Exponential decay fitting of the delay curve

Acoustic signals at different frequencies (0.7, 6, and 10 kHz) with varying intensities (from 0.01 to 0.44 Pa) are applied to the coupling system. The output of the sound level meter and the response amplitude of the OSC are collected separately and fitted linearly, as shown in Fig. 4a. The error bars are added to the sensitivity fitting graph (Supplementary Tables S4, 5, and 6). However, since the error is relatively small and the response voltage amplitude is relatively large, the error is not obvious in the figure (Supplementary Figs. S10, 11, and 12). Due to the parameter limits of the PD, the maximum response amplitude is 3.6 V. The results show that the deformation of the CaF_2_ resonator acoustic sensing structure increases linearly with increasing acoustic pressure at different frequencies before reaching the saturation voltage, and the deformation is the largest at 10 kHz. The transmission intensity is modulated by the acoustic pressure and shows a sinusoidal waveform in the time domain (Supplementary Fig. S8). A linear fit of the acoustic pressure and the response amplitude shows a linear response, and the slope is the acoustic pressure sensitivity of 11.54 V/Pa with a linearity error of 0.03%. The sensitivity at different frequencies is shown in Supplementary Fig. S9.

The minimum detectable acoustic pressure (MDP) is calculated based on the following equation.

4 $${\rm{MDP}}=\sqrt{\frac{\tau }{\rm{SNR}}}\times {P}_{\rm{applied}}$$

where *τ* is the signal duration time, BW is the spectrum analyzer resolution bandwidth, and SNR is the signal-to-noise ratio.

By varying the acoustic pressure (*P*_applied_) and the reference BW, different SNRs and MDPs can be obtained, as shown in the inset of Fig. 4b (Supplementary Table S7). The noise floor of the system is shown as the red line in Fig. 4b, and the blue line is the response signal on the ESA when *P*_applied_ is 0.31 Pa. When the *SNR* is 56.4 dB, and the BW is 50 Hz, the MDP for the resonator is calculated to be 9.4 µPa/Hz^1/2^. With reference to an acoustic pressure of 20 µPa/Hz^1/2^ in air, the minimum detectable acoustic pressure is −6.56 dB. When *Q* is 1.02 × 10^8^, the maximum detectable acoustic pressure is 0.31 Pa, which is equivalent to an acoustic pressure of 83.86 dB. Consequently, the dynamic response range of the proposed sensor is 90.41 dB.

When the loaded signal disappears, the CaF_2_ resonator does not immediately return to its original state but still continues to vibrate for a period of time, similar to a traditional tuning fork. The reasons are mainly divided into the following two aspects. On the one hand, the loaded signal collides with air molecules during oscillation, resulting in a viscous damping loss. On the other hand, it is caused by the clamping loss of its own structure. Presumably, the second cause contributes more to our sensing system. The response curves of the loudspeaker source and the CaF_2_ resonator are recorded simultaneously by OSC at 10 kHz, as shown in the blue and red lines of Fig. 4c, respectively. When the loaded signal disappears, the loudspeaker shuts off immediately without any delay, but the intensity of the CaF_2_ resonator gradually weakens with time until it finally disappears. This represents a ring-down shape, indicating the delay phenomenon of the resonator. The delay curve of the resonator is fitted with exponential decay, and the delay time (*τ*) is 0.04 s, as shown in Fig. 4d. The inset is the phase diagram, which plots the first-time derivative of the transmission as a function of the transmission. This represents a state that gradually stabilizes with time. Similarly, we recorded and fitted the other frequencies accordingly and obtained the decay times at frequencies of 1–10 kHz, as shown in Supplementary Figs. S13 and S14 and Table S8. The results show that there are differences in the delay time at different frequencies, but the time is between 20 and 40 ms.

## Vectoriality and frequency response range

The vectorial of the 7-shaped CaF_2_ acoustic sensor can be expressed as:

5 $$K=20\,\mathrm{lg}\,\frac{G{^\prime} }{G}$$

where *G’* and *G* are the maximum and minimum values measured in the directivity of the vector acoustic sensor, respectively.

Vectoriality is characteristic that the sensitivity of the acoustic sensor changes with the direction of the acoustic wave, which is usually represented by a directivity diagram, and it is also a unique feature that distinguishes the vector acoustic sensor from the scalar acoustic sensor. When measuring the directional characteristics of the vector acoustic sensor, the rotating device drives the loudspeaker to rotate 360° along the horizontal axis, simultaneously measures the sensitivity of the vector sensor in all directions, and finally obtains the directional diagram of the vector sensor.

**Fig. 5:**
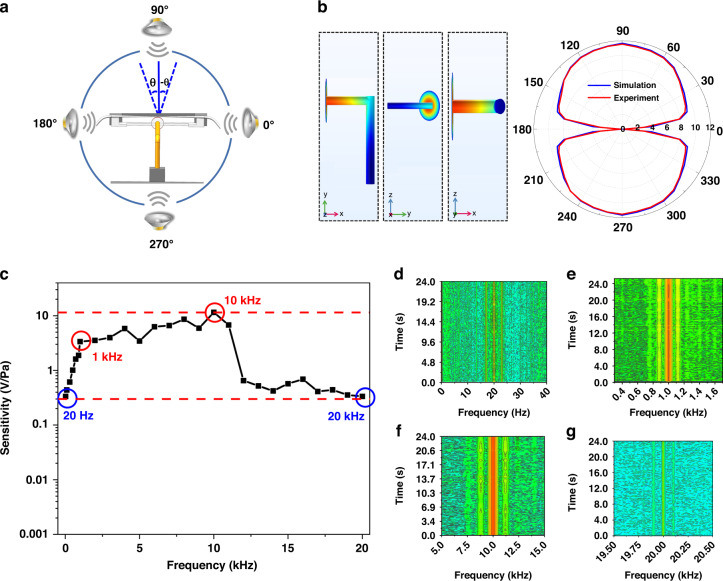
Characterization of the vectorial and frequency response of the sensor. **a** Schematic diagram of vector testing. **b** Directivity diagram of the 7-shaped CaF_2_ resonator structure at 10 kHz obtained by a simulation and experiment. **c** The frequency response curve of the proposed sensor. **d**–**g** Frequency response of the acoustic sensor at 20 Hz, 1 kHz, 10 kHz, and 20 kHz frequencies

Figure 5a shows the schematic setup of the vector acoustic sensing system. An acoustic signal at 10 kHz is used as the excitation source, and the loudspeaker is located on the side of the 7-shaped CaF_2_ resonator coupling system at 0°. We first use the FEM to simulate the vector property of the system, as shown on the left of Fig. 5b, and the directivity is calculated to be 40.94 dB. Different frequencies (900 Hz, 1 kHz, and 6 kHz) are shown in Supplementary Fig. S17. The loudspeaker is rotated around the vertical axis of the center of the CaF_2_ resonator at 10° increments, and the incident angle and the sensitivity are recorded in the experiment, as illustrated in the red line of Fig. 5b. The measured directional map shows that the proposed sensor has a vectorial property of 36.4 dB in the range of 0–360° at 10 kHz. There are certain errors between the experimental results and the simulation results. The main reason is that in the experimental test, the accuracy of the rotating device and various fixed devices is not sufficient, and various instruments and environmental noise also affect the experimental results. However, the changing trend of the experimental directionality of the acoustic sensor system is consistent with the simulation results.

Sensitivity can only show the response amplitude of the resonator to different acoustic pressure signals but cannot measure the frequency range of the 7-shaped CaF_2_ resonator. Therefore, it is necessary to test the frequency response range of the 7-shaped CaF_2_ resonator. Acoustic signals with fixed amplitude and frequencies between 20 Hz and 20 kHz produced by the loudspeaker are applied to the sensor, while the sensitivity of the acoustic signal response at different frequencies is recorded. Figure 5c shows the sensor frequency response over the range from 20 Hz to 20 kHz, which exhibits a dominant resonance peak at approximately 10 kHz. Figure 5d–g corresponds to the circles in Fig. 5c, and the frequencies are 20 Hz, 1 kHz, 10 kHz, and 20 kHz, respectively, where the depth of the color represents the magnitude of the amplitude. In the spectrum domain, a sideband appears on each side of the center frequency, which is 120 Hz different from the center frequency. This should be caused by the sinusoidal modulation of the system by the phase modulator, which needs to be further studied.

## Acquisition and reconstruction of speech signals

**Fig. 6:**
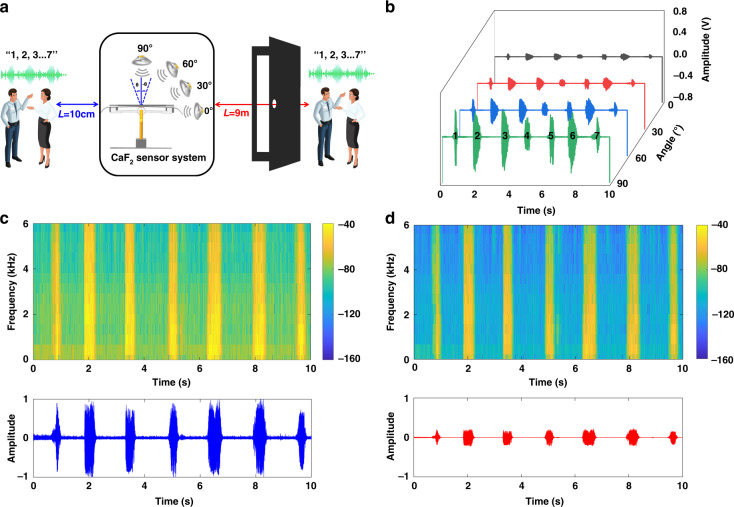
Evaluation of the performance of the sensor system. **a** Schematic diagram of the speech acquisition test at different distances (10 cm and 9 m). **b** Response curve of the sensor at 0–90°. **c**, **d** Time-domain and spectrogram diagrams of the signal reconstructed by the CaF_2_ system at distances of 10 cm and 9 m, respectively

Due to the vector property of the proposed sensor system, the response of the sensor is different at various angles. To ensure consistency at different positions, the audio clip of the sentences “1, 2, 3, 4, 5, 6, 7” in Chinese is recorded, which is almost the same as the decibel of a human speaking voice. The audio is applied at 30° increments in the horizontal plane, as illustrated in the inset of Fig. 6a. The sensor responses are recorded and plotted at various angles in Fig. 6b. The response amplitude is the largest at 90°, and as the angle gradually decreases, the voltage amplitude response also gradually decreases. The experimental result of the measured directional map in Fig. 6b shows that the proposed sensor is effectively directional.

To evaluate the weak signal detection capability of the proposed acoustic sensing system, the audio is broadcast at 10 cm and 9 m (including the effect of the wall as an obstacle) from the center of the system, corresponding to near-field and far-field tests, respectively. The acoustic response data of the sensor system at different distances are collected, as shown in Fig. 6a. The experiment is carried out in a clean room to reduce the interference of the external environment, such as dust and noise, so that the prepared CaF_2_ resonator can be stored for a long time, and the *Q* does not drop significantly. In the experiment, the recording duration is 10 s, and the recorded data of the sensor at different distances are reconstructed. Figure 6c, d shows the time-domain and spectrogram diagrams of the reconstructed acoustic signal, respectively. Due to the ultrahigh sensitivity and SNR, good linearity, and broadband frequency of the sensor system, the reconstructed audio signal can correspond to each beat of the analog sound signal, and the sensor system can still clearly display the music characteristics at a distance of 9 m. The experimental results fully verify the ultrahigh sensitivity and weak signal detection capability of the system.

## Precise speech recognition and separation

**Fig. 7:**
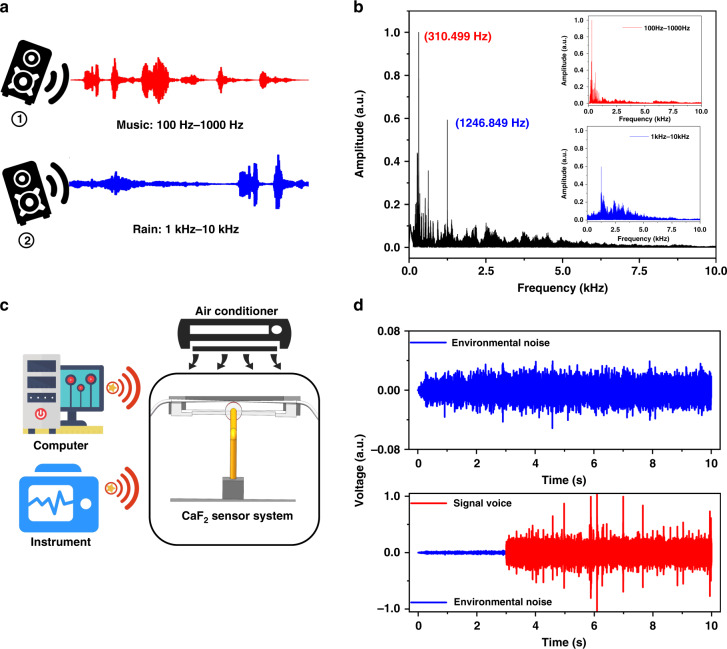
Application of the CaF_2_ resonator sensing system in speech recognition and separation. **a** Experimental setup for the simultaneous playing of different music segments. **b** The frequency-domain diagram of the signal reconstructed by the CaF_2_ system. **c** Schematic diagrams of various background noises. **d** The voltage comparison between environmental noise and signal voice

The CaF_2_ resonator acoustic sensor system can not only detect weak signals but also accurately identify and separate multiple sounds. Specifically, two different music pieces act simultaneously on the center of the sensing system to verify its ability to separate different sound signals. Figure 7a shows the case of the simultaneous playing of music segments with different frequencies, one of which is mainly concentrated in ① (100–1000 Hz) and the other in ② (1–10 kHz). The frequency response diagram collected by the CaF_2_ resonator sensing system is shown in Fig. 7b. The relatively low frequency is generated by ①, while the signal with a relatively high frequency is generated by ②. Therefore, the two simultaneous sounds can be identified and separated.

The CaF_2_ resonator sensor system shows better sensitivity to high-frequency sound than lower-frequency sound, which is convenient for using different voltage responses for separation. Our experiment is completed in an ultraclean room, which contains a variety of different sound sources, including (i) the working noise of various instruments, (ii) the sound of air conditioning, and (iii) the sound of a relatively high-frequency rain signal, as shown in Fig. 7c. Since the rain signal is much greater than the background noise, the voltage can be used to separate the signal from the environmental noise. The time-domain signal obtained after separation is shown in Fig. 7d, and the signal voice is approximately 15 times larger than the environmental noise. Thus, we verify that in a laboratory with background noise, the CaF_2_ resonator sensor system can still complete the separation of multiple sound sources.

## Conclusion

In this work, we demonstrate an ultrahigh-sensitivity acoustic sensor system based on an ultrahigh-*Q* CaF_2_ resonator. When the radius of the resonator is changed from 2.0 to 5.0 mm, and the thickness is reduced from 1.0 to 0.1 mm, the change in the resonator radius caused by the acoustic pressure increases to 1.13 × 10^−^^7^ mm/Pa. The corresponding resonance wavelength shift is also increased by more than two orders of magnitude, which is 3.5 × 10^−5^ nm/Pa. Combined with the frequency locking technique, the sensitivity can easily reach 11.54 V/Pa at a frequency of 10 kHz when *Q* is selected to be 1.02 × 10^8^, which is higher than that of other optical resonator acoustic sensors. Meanwhile, the minimum detectable acoustic pressure level of the proposed system is as low as 9.4 µPa/Hz^1/2^, which greatly improves the detection resolution. With a good directionality of 36.4 dB and broadband frequency response range of 20 Hz–20 kHz, the sensor system can not only achieve long-distance (9 m) speech signal acquisition and reconstruction with a wall as an obstacle but also accurately identify and separate multiple voices in noisy environments. The excellent performance of the device will have great application potential in weak signal detection.

## Supplementary information

Supplementary information
