# Supplementary material for: An ultrahigh sensitivity acoustic sensor system for weak signal detection based on an ultrahigh-Q CaF2 resonator
Source: Microsyst Nanoeng. 2023 May 17;9:65. doi: 10.1038/s41378-023-00540-0 (PMC10192424; doi:10.1038/s41378-023-00540-0)
Supplement: Supplementary file 1 — Supplementary information [file 41378_2023_540_MOESM1_ESM.docx]

**Supplementary information**

**An ultrahigh sensitivity acoustic sensor system for weak signal detection based on an ultrahigh-*Q* CaF_2_ resonator**

Tong Xing^1^, Enbo Xing^1*^, Tao Jia^2^, Jianglong Li^1^, Jiamin Rong^2^, Li Li^3^, Sicong Tian^4^, Yanru Zhou^1^, Wenyao Liu^1^, Jun Tang^2*^, and Jun Liu^1*^

^1^Key Laboratory of Dynamic Testing Technology, School of Instrument and Electronics, North University of China, Taiyuan 030051, China.

^2^School of Semiconductors and Physics, North University of China, Taiyuan 030051, China.

^3^Shanxi Key Laboratory of Advanced Semiconductor Optoelectronic Devices and Integrated Systems, Jincheng, 048026, China.

^4^State Key Laboratory of Luminescence and Applications, Changchun Institute of Optics, Fine Mechanics and Physics, Chinese Academy of Sciences, Changchun 130033, China

Correspondence: Enbo Xing, Jun Tang and Jun Liu ([xiaoxing1228@126.com](mailto:xiaoxing1228@126.com); [tangjun@nuc.edu.cn](mailto:tangjun@nuc.edu.cn); and [liuj@nuc.edu.cn](mailto:liuj@nuc.edu.cn))

This file includes:

- Method

The details of the coupling between the tapered fiber and resonator.

- Simulation and experimental data:

1. Simulation details for the software name and simulation procedure.
2. Simulation of the displacement modes and frequency response of 7-shaped CaF_2_ resonator.
3. The effect on the performance of the copper column.
4. Derivation of coupled modal theory equations driven by acoustic elastic waves.
5. The sensitivity and error bars at different frequencies in the experiment.
6. Optimization of the minimum detectable acoustic pressure.
7. The delay response curve of the CaF_2_ resonator at different frequencies.
8. Vectoriality of the 7-shaped CaF_2_ resonator at different frequencies.

**Method**

**The details of the coupling between the tapered fiber and resonator.**

In the actual experimental process, we build a set of optical coupling test platform, including two CCD cameras, which are used to observe the coupling state of the upper and side respectively, as shown in Fig. S1. During the experiment, the resonator and the tapered fiber are placed on the top of two six-dimensional adjusting frames respectively to achieve fine adjustment. In order to better achieve coupling test, we also use 650 nm red light meter for auxiliary observation to better judge the coupling state between the resonator and the tapered fiber. The tapered fiber is attached to the surface of the CaF_2_ resonator, that is over-coupled, which not only avoids the influence of environmental fluctuation noise, but also eliminates the influence of the dissipative response regime. But at the same time, over-coupled will also affect the coupling efficiency of the CaF_2_ resonance spectrum, resulting in coupling loss.


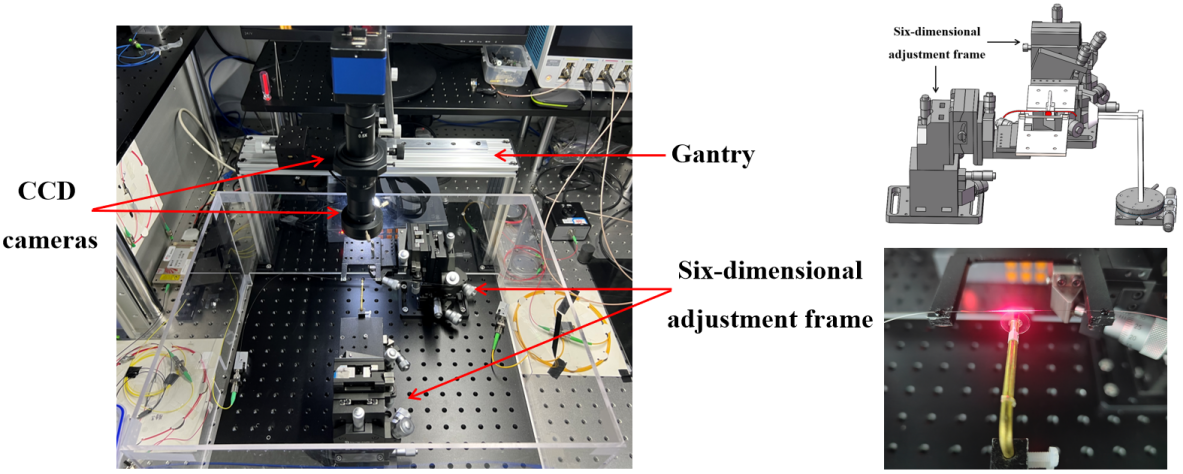


**Fig. S1.** The details of the coupling between the tapered fiber and the resonator.

In order to illustrate the coupling between the tapered fiber and the resonator more clearly, we provide the micrograph, as shown in Fig. S2. The radius of the tapered fiber is 1.8 μm.


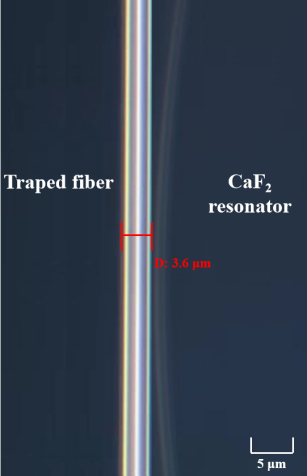


**Fig. S2.** The micrograph about the coupling between the tapered fiber and the resonator.

1. Simulation details for the software name and simulation procedure.

In the simulation procedure, the name of the commercial software we used is COMSOL Multiphysics 5.6. The main feature of this software is that it can perform multi-physical field coupling analysis, which can well simulate the force situation of complex structures under different environments, verify the rationality of the structure and optimize it. When analyzing the structural characteristics of the CaF_2_ resonator, such as characteristic frequency and stress distribution, we use the structural mechanics module. When analyzing the optical field characteristics of the CaF_2_ resonator, we use the Wave optics. When analyzing the response characteristics of the CaF_2_ resonator to the acoustic signal, the acoustic module and the structural mechanics module are coupled.

The details of the specific simulation process are as follows: firstly, the geometric structure model of the CaF_2_ resonator needs to be constructed, including the radius (*R*) and thickness (*H*) of the CaF_2_ resonator and supporting copper column. Secondly, the material properties of each part need to be defined, the resonator is set to the material parameters of CaF_2_, the column supporting the resonator is set to the material properties of copper, and the outermost sphere is to simulate the air domain, using the built-in material air as its material property. The specific settings and material parameters are shown in Table S1^1,2^. Immediately after, the physical field needs to be set up. In the Solid Mechanics physical field, the bottom of the copper column used to support the CaF_2_ resonator is fixed; in the Pressure Acoustics, Frequency Domain physical field, the Spherical Wave Radiation boundary condition can be well used to simulate the propagation of acoustic waves very well. The globally defining parameters and the boundary condition are as shown in Table S2.

**Table S1.** Material parameters of the CaF_2_ resonator and copper column.

| **Material parameters of CaF_2_ resonator and copper column** | | |
| --- | --- | --- |
|  | **CaF_2_ resonator** | **Copper column** |
| **Density (kg/m³)** | **3180** | **8900** |
| **Young’s Modulus (Gpa)** | **75.8** | **110** |
| **Poisson Ratio** | **0.26** | **0.34** |
| **Radius (mm)** | **2.0 mm~5.0 mm** | **1.0 mm** |
| **Thickness (mm)** | **0.1 mm~1.0 mm** | **10.0 mm** |

**Table S2.** Globally defining parameters and the boundary condition.

| **Simulation setup** | **Global**  **variables** | **Physical quantity** | **Value** | **Describe** |
| --- | --- | --- | --- | --- |
|  |  | ***k*_1_** | **sin(θ)*cos(φ)** | **Incident wave direction vector, x component** |
|  |  | ***k*_2_** | **sin(θ)*sin(φ)** | **Incident wave direction vector, y component** |
|  |  | ***k*_3_** | **cos(θ)** | **Incident wave direction vector, z component** |
|  | **Boundary conditions** | **Physical quantity** | **Value** | **Describe** |
|  |  |  | **(sin(θ)*cos(φ), sin(θ)*sin(φ), cos(θ))** | **Incident wave direction vector** |
|  |  | ***p*_0_** | **1 Pa** | **Pressure amplitude** |

After the physical field is defined, the entire simulation model is meshed for dissection, and the minimum cell size of the mesh should be less than 1/6 of the minimum size in the structure to ensure the accuracy of the calculation. When we choose the physical field control grid for the grid dissection, COMSOL will automatically dissect the grid according to the physical field settings in the model, and the user can manually select nine preset grid size levels, as shown in Table S3. The physical field control grid can meet most of the computational needs, and when the quality of the grid formed by the physical field control grid is low, the user can manually control the grid on the basis of the grid formed by the physical field control grid to further optimize the grid profile. Finally, add the study and perform the frequency domain calculations.

**Table S3.** COMSOL grid division settings.

| **Mesh** | **User-controlled grid** | **Physical quantity** |
| --- | --- | --- |
|  |  | **Free triangular grid** |
|  |  | **Free quadrilateral grid** |
|  | **Physical field control grid** | **Physical quantity** |
|  |  | **nine preset grid size levels**  **(extremely-fine, ultra-fine, more fine, fine, conventional, coarse, more coarse, ultra-coarse, extremely-coarse)** |

1. Simulation of the displacement modes and frequency response of 7-shaped CaF_2_ resonator.

The displacement at different resonance frequencies of the 7-shaped CaF_2_ resonator sensor system is simulated using the FEM. The frequency response from 20 Hz to 20 kHz and the corresponding displacement modes at 20 Hz, 1 kHz, 10 kHz and 20 kHz have been shown in the main text. The frequencies with relatively large displacement changes are selected, and their displacement modes are supplemented in Fig. S3.


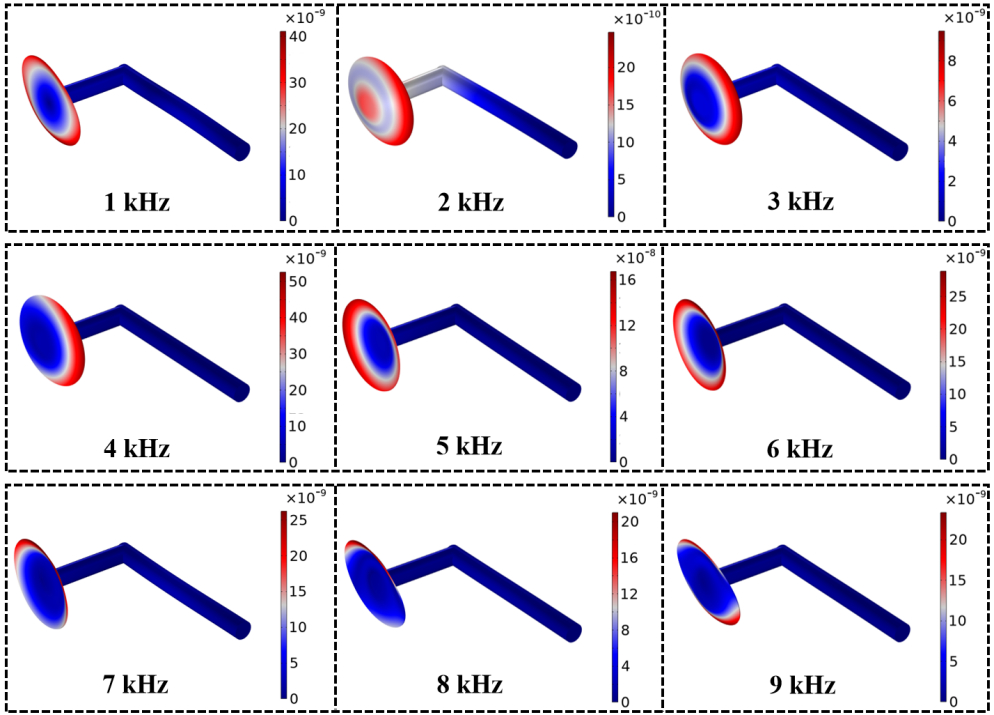


**Fig. S3.** The corresponding displacement modes at different frequencies.

During the finite element method (FEM) simulation, when the frequency is lower than 20 Hz or higher than 20 kHz is also simulated, as shown in Fig. S4, and it can be seen that when the frequency is lower than 20 Hz, the response of the resonator decreases significantly, and when the frequency is higher than 20 kHz, the response of the resonator gradually flattens.

**Fig. S4.** The frequency response characteristic when the frequency is higher than 20 Hz or higher than 20 kHz.

1. **The effect on the performance of the copper column.**

In the manuscript, the role of the copper column is to support CaF_2_ resonator for the later experimental coupling tests. By using FEM simulation, we analyze the effect of different sizes of the copper column and different material parameters on the sensing system, respectively.

Firstly, the effect of different copper column sizes on the sensing system. By modifying the radius of the copper column (3 mm, 2.5 mm, 2 mm, 1.5 mm, 1 mm), different deformation variables can be obtained, and the results are shown in Fig. S5. It can be seen that with the decrease of the copper column radius, the deformation variations of the sensor system increase rapidly, and the corresponding sensitivity improved higher. According to the actual experimental conditions, we finally choose the radius of copper column is 1 mm.

**Fig. S5.** The change of radius under different radius of copper column.

Secondly, we also analyze the influence of different materials on the sensing system. Since different materials have different Young’s modules, and the results are shown in Fig. S6. As can be seen from the figure, the influence of different materials on the deformation variables of the system is not much different, which indicates that the influence of replacing the support materials with different materials on the system is negligible.

**Fig. S6.** Relationship between Young’s modulus and the change of radius of different materials.

In summary, the smaller the radius of the copper column used to support the CaF_2_ resonator, the larger the deformation variable of the system and the higher the corresponding sensitivity. However, due to the limitations of the actual experimental test conditions, the size of the copper column cannot be infinitely small. In addition, copper is relatively soft and easy to process, which can be transformed into different sizes according to our experimental requirements. It has been shown above that different materials have very little effect on the system, therefore, we choose copper as our support material.

1. **Derivation of coupled modal theory equations driven by acoustic elastic waves.**

From the perspective of physical mechanism, acoustic sensing based on resonator mainly comes from two mechanisms: dispersive coupling response and dissipative coupling response. The dispersive coupling response is that the acoustic wave modulates the refractive index and geometric morphology of the resonator through mechanical effects, resulting in the resonance frequency shift of the resonator. The dissipative coupling response is the change of coupling conditions of the resonator through acoustic wave modulation, leading to the change of coupling loss, which broadens or narrows the transmission spectrum linewidth. In the experiment, the tapered fiber is attached to the surface of the CaF_2_ resonator, which eliminates the influence of the dissipative response regime.

Using coupled-mode theory, the optical mode amplitude a is expressed by^3.4^:

$\frac{da(t)}{dt}=(i(\Delta+Gsin(\omega_{m}t))-\frac{\kappa}{2})a(t)+\sqrt{\kappa_{e}}a_{in}$ (S1)

where Δ=*ω_L_*-*ω_r_* is the detuning of the laser from the optical resonance, *G* represents the resonance shift due to acoustic pressure, *κ* is the overall intensity decay rate, *κ_e_* represents the input coupling losses, *a*_in_ is input optical field into the resonator. *ω*_m_ is the acoustic frequency.

The output field of the resonator follows the input-output relationship: $a_{out}=-a_{in}+\sqrt{\kappa_{e}}a$. The normalized transmission can therefore be expressed as T=|*a*_out_/*a*_in_|^2^:

$T=\left|-1+\frac{\sqrt{k_{e}}a}{a_{in}}\right|^{2}$ (S2)

The transmission therefore changes with acoustic pressure, as shown in the left of Fig. S7. As the acoustic pressure gradually increases, the frequency shift also increases gradually. The normalized transmission change with respect to the resonance shift derived by the first derivative of the transmission spectrum (*dT/df*) in the right of Fig. S7.


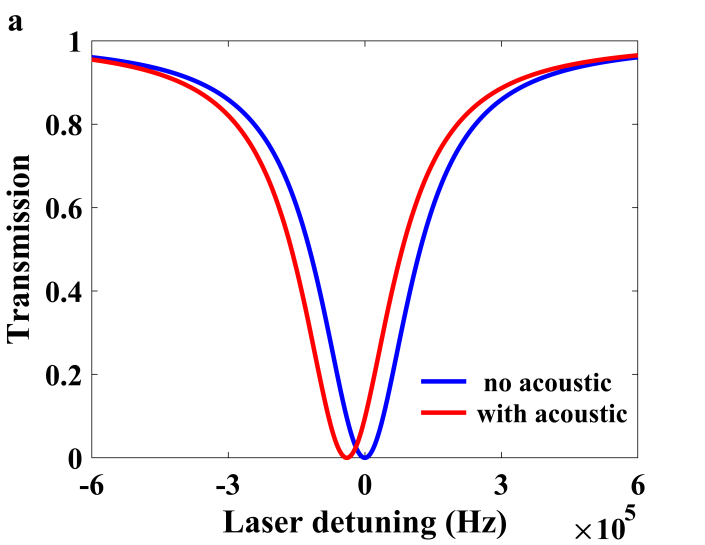

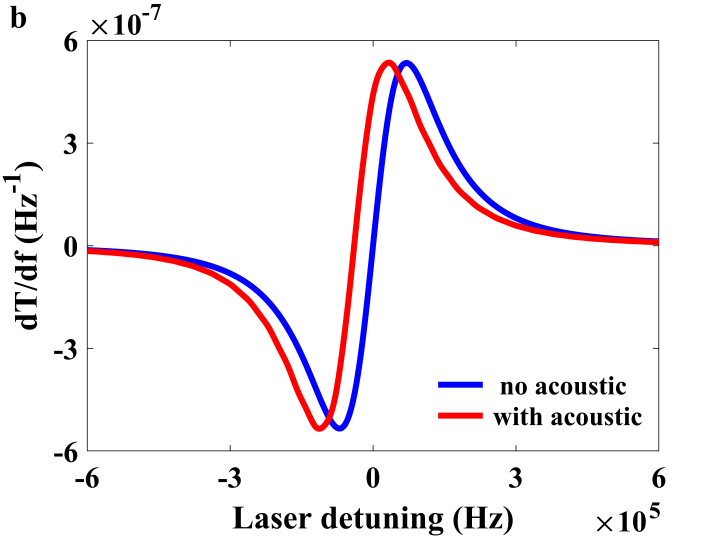


**Fig. S7.** The frequency shift and *dT*/*df* changes with the acoustic pressure.

The sensitivity of the CaF_2_ resonator acoustic sensor system can be defined as:

$S=\frac{dT}{dP}=\frac{d\lambda_{r}}{dP}\frac{dT}{d\lambda_{r}}\text{=}\frac{\lambda_{r}}{dP}\frac{dR}{R}\frac{dT}{d\lambda_{r}}$ (S3)

where *T* is the transmission, *P* is the acoustic pressure, λ_r_ is the resonance wavelength, *dT/dλ_r_* is defined as the slope of its linear region, which can be approximated as linearly proportional to the *Q* factor.

Based on the above analysis, *G* is very important, and it is obtained by experimental tests. The dispersive coupling response is that when the acoustic pressure acts on the CaF_2_ resonator, the *R* and *n_eff_* of the resonator will change, which will lead to the resonance shift and affect the detuning term of the dispersion coupling equation and becomes *Δ+Gsin(ω_m_t)*. In the experiment, we obtain the sensitivity of the sensing system by testing, and the resonance shift is obtained according to the Eq. S3. When the frequency is 10 kHz, the sensitivity is 11.54 V/Pa, and the *G* is calculated to be 3.99 MHz/Pa. Similarly, the *G* is calculated to be 2.18 MHz/Pa and 0.56 MHz/Pa when the frequencies are 6 kHz and 0.7 kHz, respectively.

1. **The sensitivity and error bars at different frequencies in the experiment.**

The transmission intensity is modulated by the acoustic pressure and shows a sinusoidal waveform in the time domain. The outputs from the sound level meter and the response amplitude are collected separately, as shown in Fig. S8. The results show that the response amplitude increases linearly with acoustic pressure before reaching the saturation power of 3.6 V. A linear fit of the acoustic pressure and the response amplitude, showing a linear response, the slope is the acoustic pressure sensitivity of 11.54 V/Pa with the linearity error of 0.03%.


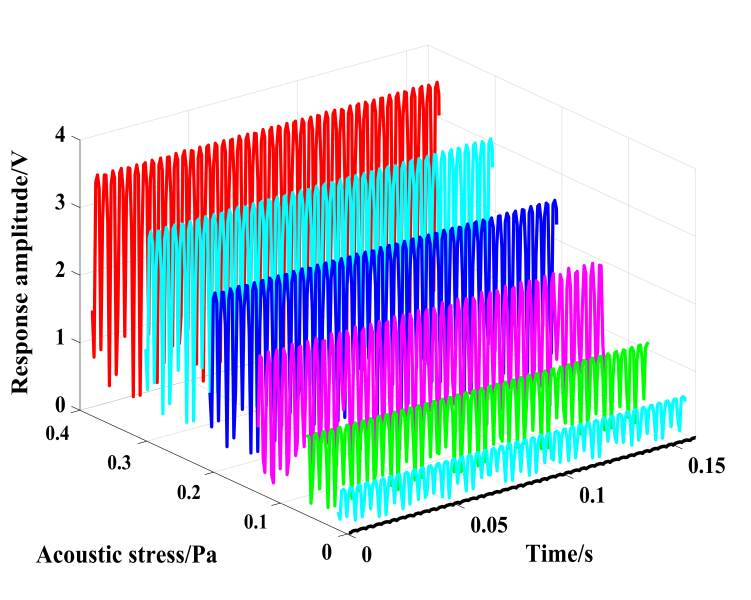


**Fig. S8.** The transmission spectrum in time domain and linear fitting changes with the acoustic pressure at 10 kHz.

The sensitivity at different frequencies (0.7 kHz, 6 kHz) is shown in Fig. S9. Due to the parameter limitation of the PD, the maximum response amplitude is 3.6 V. A linear fit of the acoustic pressure and the response amplitude, the sensitivity is 1.61 V/Pa at 0.7 kHz and 6.29 V/Pa at 6 kHz.

**Fig. S9.** The sensitivity at different frequencies.

In the experiment, when the frequency of the acoustic signal is constant, the response voltage amplitude with the acoustic pressure on the oscilloscope is recorded. Under the same acoustic pressure, five groups of data are tested respectively, and the mean value and standard deviation of the data are calculated as shown in Table S4, 5 and 6. In order to see the variation of errors more visually, equally spaced bar graphs are drawn to avoid the overlap of acoustic pressures with each other. The following three Fig. S10, 11 and 12 show the experimental data and the mean and standard deviation of the data at 0.7 kHz, 6 kHz and 10 kHz frequencies, respectively.

**Table S4.** The experiment data at 0.7 kHz frequency.

| **Acoustic pressure (Pa)** | **Response amplitude (mV)** | | | | | **Mean** | **Standard deviation** |
| --- | --- | --- | --- | --- | --- | --- | --- |
| **0.00893** | **10** | **9** | **11** | **8** | **14** | **10.4** | **2.30217** |
| **0.03991** | **65** | **62** | **69** | **60** | **70** | **65.2** | **4.32435** |
| **0.06325** | **100** | **105** | **103** | **99** | **102** | **101.8** | **2.38747** |
| **0.09463** | **160** | **165** | **165** | **163** | **159** | **162.4** | **2.79285** |
| **0.14656** | **236** | **231** | **242** | **250** | **221** | **236** | **10.97725** |
| **0.22963** | **379** | **386** | **397** | **365** | **372** | **379.8** | **12.39758** |
| **0.30622** | **489** | **474** | **493** | **480** | **479** | **483** | **7.77817** |
| **0.31698** | **500** | **496** | **501** | **497** | **506** | **500** | **3.937** |
| **0.35566** | **568** | **570** | **582** | **562** | **581** | **572.6** | **8.6487** |
| **0.39905** | **640** | **644** | **653** | **640** | **635** | **642.4** | **6.73053** |


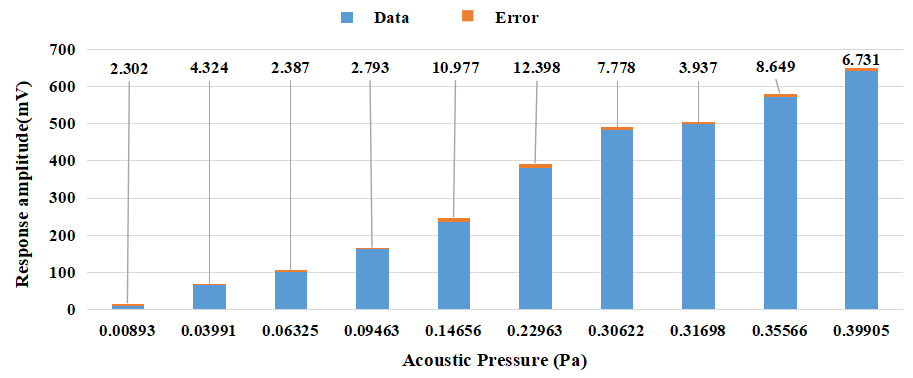


**Fig. S10.** Bar chart of error at 0.7 kHz frequency.

**Table S5.** The experiment data at 6 kHz frequency.

| **Acoustic pressure (Pa)** | **Response amplitude (mV)** | | | | | **Mean** | **Standard deviation** |
| --- | --- | --- | --- | --- | --- | --- | --- |
| **0.00893** | **54** | **53** | **56** | **58** | **60** | **56.2** | **2.86356** |
| **0.03991** | **250** | **246** | **246** | **253** | **260** | **251** | **5.83095** |
| **0.06325** | **385** | **377** | **392** | **395** | **390** | **387.8** | **7.04982** |
| **0.09463** | **598** | **586** | **598** | **608** | **586** | **595.2** | **9.33809** |
| **0.14656** | **920** | **913** | **926** | **930** | **920** | **921.8** | **6.49615** |
| **0.22963** | **1480** | **1470** | **1492** | **1500** | **1480** | **1484.4** | **11.69615** |
| **0.30622** | **1921** | **1914** | **1930** | **1931** | **1934** | **1926** | **8.27647** |
| **0.31698** | **1990** | **1986** | **2002** | **1999** | **1992** | **1993.8** | **6.57267** |
| **0.35566** | **2248** | **2246** | **2264** | **2263** | **2264** | **2257** | **9.16515** |
| **0.39905** | **2500** | **2508** | **2521** | **2498** | **2523** | **2510** | **11.59741** |


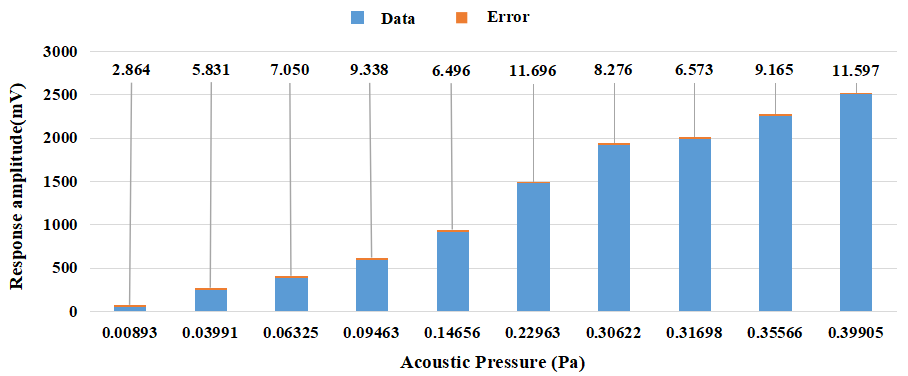


**Fig. S11.** Bar chart of error at 6 kHz frequency.

**Table S6.** The experiment data at 10 kHz frequency.

| **Acoustic pressure (Pa)** | **Response amplitude (mV)** | | | | | **Mean** | **Standard deviation** |
| --- | --- | --- | --- | --- | --- | --- | --- |
| **0.00893** | **87** | **84** | **86** | **88** | **90** | **87** | **2.23607** |
| **0.03991** | **476** | **485** | **470** | **480** | **464** | **475** | **8.24621** |
| **0.06325** | **752** | **740** | **763** | **768** | **742** | **753** | **12.40967** |
| **0.09463** | **1097** | **1092** | **1100** | **1103** | **1093** | **1097** | **4.63681** |
| **0.14656** | **1715** | **1712** | **1702** | **1721** | **1725** | **1715** | **8.86002** |
| **0.22963** | **2592** | **2587** | **2595** | **2599** | **2587** | **2592** | **5.19615** |
| **0.30622** | **3553** | **3540** | **3556** | **3565** | **3546** | **3552** | **9.56556** |
| **0.31698** | **3581** | **3575** | **3586** | **3589** | **3574** | **3581** | **6.59545** |
| **0.35566** | **3599** | **3589** | **3616** | **3586** | **3605** | **3599** | **12.18606** |
| **0.39905** | **3610** | **3584** | **3586** | **3602** | **3618** | **3600** | **14.8324** |


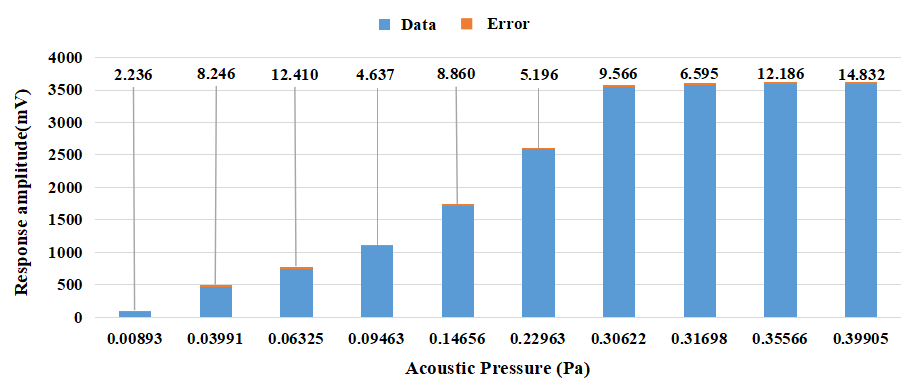


**Fig. S12.** Bar chart of error at 10 kHz frequency.

1. **Optimization of the minimum detectable acoustic pressure**

The minimum detectable acoustic pressure (*MDP*) is calculated based on the following equation.

$MDP=\sqrt{\frac{\tau}{SNR}}\times P_{applied}$ (S4)

where *τ* is the signal duration time of *τ* = *BW*^−1^, *BW* is the spectrum analyzer resolution bandwidth, *SNR* is the signal-to-noise.

By varying the acoustic pressure (*P_applied_*) and the reference *BW* for ESA, the different SNR and MDP can be obtained, as shown in Table S7. When the *SNR* is 56.4 dB and *BW* is 50 Hz, the *MDP* for the resonator is calculated to be 9.4 µPa/Hz^1/2^.

**Table S7.** The minimum detectable acoustic pressure at different *SNR*, acoustic pressure and *BW*.

| **The minimum detectable acoustic pressure (MDP)** | | | | |
| --- | --- | --- | --- | --- |
| **BW (Hz)** | **SNR (P=0.31 Pa)** | **SNR (P=0.25 Pa)** | **SNR (P=0.16 Pa)** | **SNR (P=0.08 Pa)** |
| **1** | **66.1 dB** | **60.1 dB** | **54.2 dB** | **49.1 dB** |
| **5** | **64.2 dB** | **58.0 dB** | **52.1 dB** | **47.1 dB** |
| **10** | **62.0 dB** | **56.1 dB** | **50.0 dB** | **45.2 dB** |
| **20** | **60.1 dB** | **54.0 dB** | **48.0 dB** | **43.0 dB** |
| **30** | **58.3 dB** | **52.3 dB** | **46.2 dB** | **41.0 dB** |
| **50** | **56.4 dB** | **49.0 dB** | **43.0 dB** | **38.2 dB** |
| **100** | **50.0 dB** | **43.2 dB** | **37.1 dB** | **32.0 dB** |
| **200** | **43.1 dB** | **36.0 dB** | **30.0 dB** | **25.2 dB** |

1. **The delay response curve of the CaF_2_ resonator at different frequencies.**

The delay time dependent on frequencies is due to the effect of the mechanical oscillation characteristic of the CaF_2_ resonator. The CaF_2_ resonator itself has the inherent frequency, which is an inherent property of the structure, mainly influenced by the size, material stiffness and mass distribution. It is not related to the external excitation, and usually a structure has many inherent frequencies. When the frequency of the external applied acoustic signal is the same as the inherent frequency of the CaF_2_ resonator structure, it enhances the mechanical resonance of the structure and the deformation of the structure to reach the maximum. As can be seen from Fig. 1c and Fig. 5c in the manuscript, when the frequency is in the range of 5 kHz to 10 kHz, the mechanical deformation is large and the corresponding sensitivity is high, which is mainly caused by the mechanical resonance. When the frequency is in the range of 1 kHz to 4 kHz, the mechanical deformation and sensitivity are relatively low, the reason is that the frequency is far from the mechanical resonance, so the corresponding mechanical deformation and sensitivity are relatively low.

Since the delay time is different at different frequencies, we calculated the delay response at 1~10 kHz frequencies. At 1 kHz frequency, the response curves of the loudspeaker sound source and the CaF_2_ resonator are recorded simultaneously by oscilloscope, as shown in the blue and red line of Fig. S13, respectively. The delay curve of the resonator is fitted with exponential decay and the delay time (*τ*) is 0.025 s. The inset is the phase diagram, which is plotting the first time derivative of the transmission as a function of the transmission. Similarly, we recorded and fitted the other frequencies accordingly, and obtained the decay times at 1~10 kHz frequencies, as shown in Fig. S14. The Table S8 provides the decay times at different frequencies, indicating the time is between 20 ms~40 ms.

**Fig. S13.** a The delay response curve of the CaF_2_ resonator. b Exponential decay fitting of the delay curve.


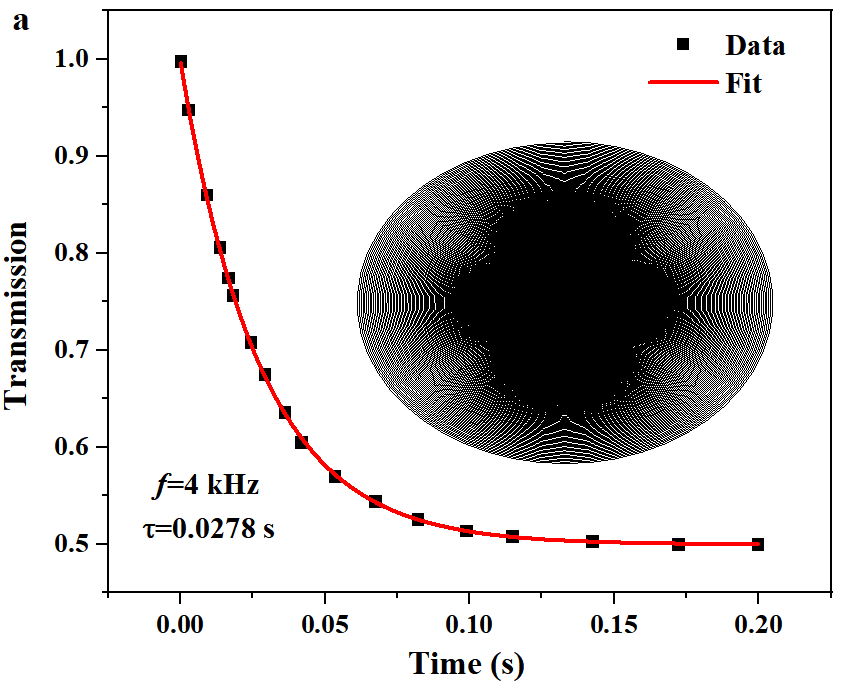

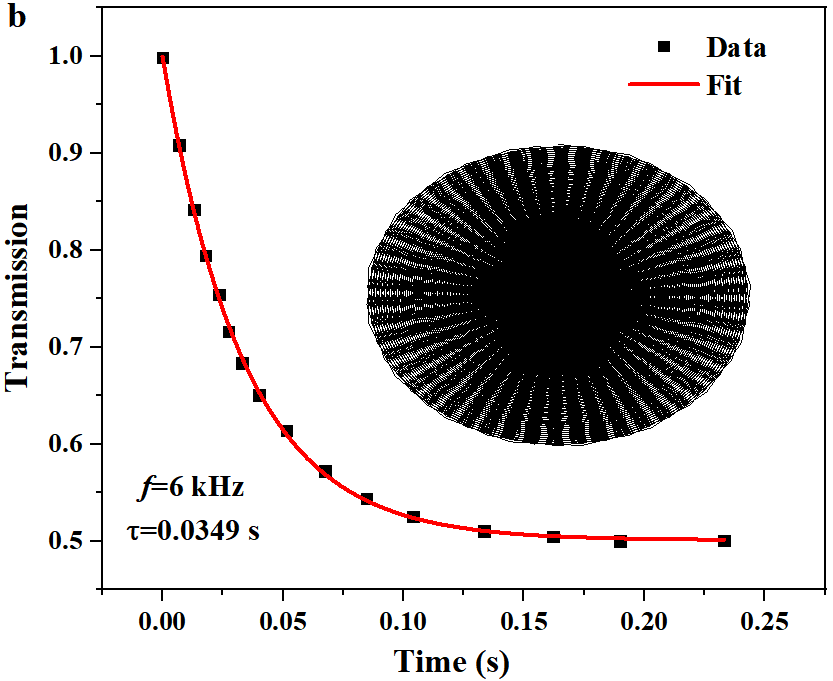


**Fig. S14.** The exponential decay fitting of the delay curve of the resonator at 4 kHz and 6 kHz.

**Table S8.** The delay time at different frequencies

| **The delay response** | | | | | | | | | | |
| --- | --- | --- | --- | --- | --- | --- | --- | --- | --- | --- |
| ***f* (kHz)** | **1** | **2** | **3** | **4** | **5** | **6** | **7** | **8** | **9** | **10** |
| **τ (s)** | **0.0247** | **0.0269** | **0.0312** | **0.0278** | **0.0356** | **0.0349** | **0.0298** | **0.0366** | **0.0347** | **0.0399** |

1. **Vectoriality of the 7-shaped CaF_2_ resonator at different frequencies.**

Vectorial property is a quantity that both magnitude and direction. In the acoustic sensor system, the CaF_2_ resonator is regarded as a circular wafer. When the acoustic pressure acts vertically on the upper surface of the resonator, the larger the deformation of the cavity is due to the larger the contact area, and the higher the sensitivity. When the acoustic pressure acts on the side of the resonator, the deformation of the resonator is almost zero, and the influence of the acoustic filed is almost negligible. By applying the acoustic signals in different directions, the deformation of resonator is not the same, which has a vectorial property, so that the signals in different directions can be distinguished. Through the above analysis, the vectorial property is derived from the natural properties of the CaF_2_ resonator.

By using finite element method (FEM) simulation, we compare this shape and other shapes, as shown in Fig. S15. During the simulation, the size of the CaF_2_ resonator and the length of the supporting rod remain unchanged, and the amplitude of the acoustic pressure is 1.0 pa. By comparing the 7 shape and other shapes, it can be seen that the influence of the different shapes of the rod on the sensing system is little, which mainly depends on the properties of the CaF_2_ resonator.


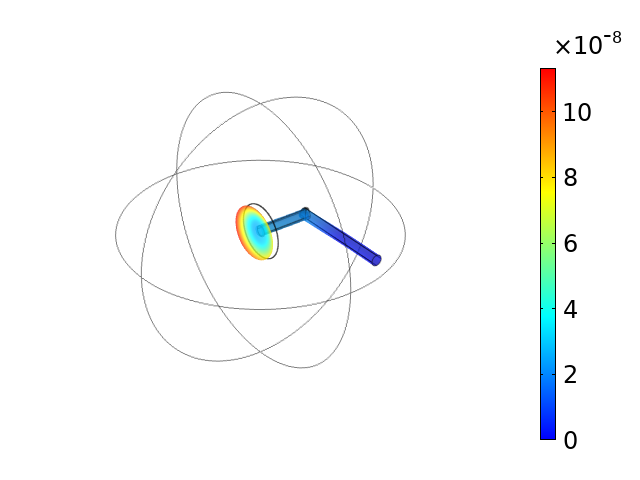

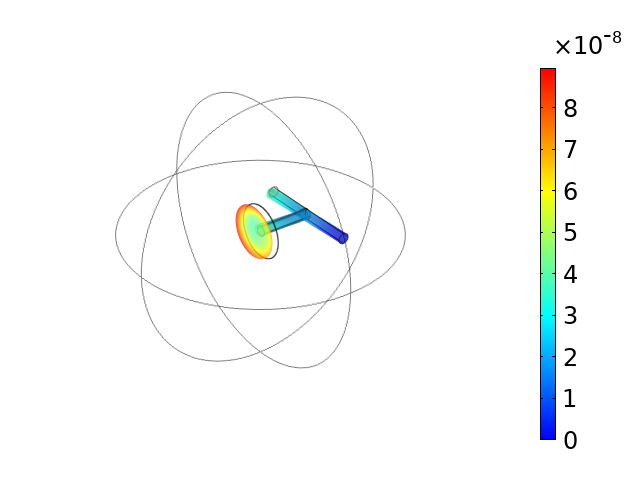

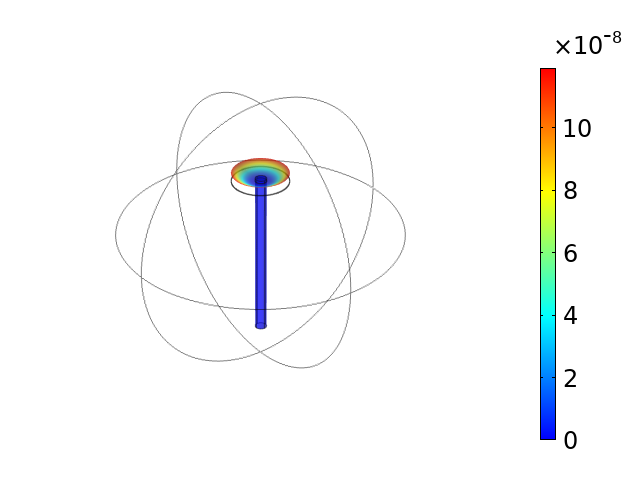


**Fig. S15.** The deformation of the sensing system with 7 shape and other shapes.

Meanwhile, we also analyze the vectoriality of the three different shapes above, which are called as 7 shape, H shape and T shape, as shown in Fig. S16. By comparing, we can see that the difference of vectoriality of the three different shapes is not much, and we choose the 7 shape for the convenience of the experimental operation.

**Fig. S16.** The vectoriality of the three different shapes.

The directivity is the characteristic that the sensitivity of the acoustic sensor changes with the direction of the acoustic wave, which is usually represented by a directivity diagram, and it is also a unique feature that distinguishes the vector acoustic sensor from the scalar acoustic sensor. Using the FEM to simulate the vector property of the system at different frequencies (900 Hz, 1 kHz and 6 kHz), as shown in Fig. S17.


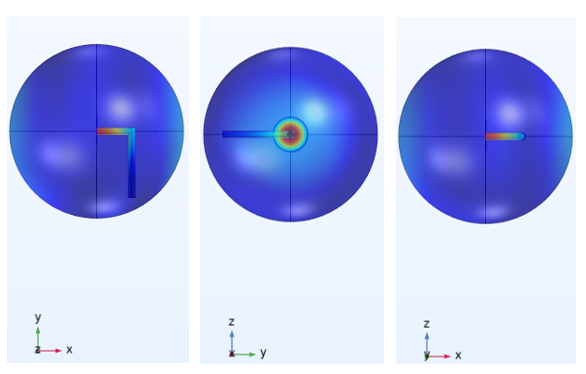


**Fig. S17.** The directivity of the 7-shaped CaF_2_ resonator at 900 Hz, 1 kHz and 6 kHz.

**REFERENCES**

1. Li, Y., Zhou, X. M., Liu, C. L. & Luo, S. N. Refractive indices of CaF_2_ single crystals under elastic shock loading *J. Appl. Phys*. **122**, 045901 (2017).
2. Xing, T. et al. Fast Switching Acoustic Sensor With Ultrahigh Sensitivity and Wide Dynamic Response Range Based on Ultrahigh-*Q* CaF_2_ Resonator. *J. Lightw. Technol.* **40**, 5775-5780 (2022).
3. Hu, Y. et al. Generation of Optical Frequency Comb via Giant Optomechanical Oscillation. *Phys. Rev. Lett.* **127**, 134301 (2021).
4. Laura, M., Martín, L. L., Griol, A., Navarro-Urrios, D. & Alejandro, M. Microwave oscillator and frequency comb in a silicon optomechanical cavity with a full phononic bandgap. *Nanophotonics* **9**, 3535-3544 (2020).
5. Li, Y. L. & Barker, P. F. Characterization and Testing of a Micro-g Whispering Gallery Mode Optomechanical Accelerometer. *J. Lightw. Technol.* **36**, 3919-3926 (2018).
